# Neuromodulatory Effects of Guanine-Based Purines in Health and Disease

**DOI:** 10.3389/fncel.2018.00376

**Published:** 2018-10-23

**Authors:** Carla I. Tasca, Débora Lanznaster, Karen A. Oliveira, Victor Fernández-Dueñas, Francisco Ciruela

**Affiliations:** ^1^Departamento de Bioquímica, Centro de Ciências Biológicas, Universidade Federal de Santa Catarina, Florianópolis, Brazil; ^2^Programa de Pós-Graduação em Bioquímica, Centro de Ciências Biológicas, Universidade Federal de Santa Catarina, Florianópolis, Brazil; ^3^UMR 1253, Team 2, INSERM/University of Tours, Tours, France; ^4^Unitat de Farmacologia, Departament de Patologia i Terapèutica Experimental, Facultat de Medicina, IDIBELL, Universitat de Barcelona, L’Hospitalet de Llobregat, Barcelona, Spain; ^5^Institut de Neurociències, Universitat de Barcelona, Barcelona, Spain

**Keywords:** guanosine, neuromodulation, purinergic system, Parkinson’s disease, Alzheimer’s disease, glutamatergic system

## Abstract

The function of guanine-based purines (GBPs) is mostly attributed to the intracellular modulation of heteromeric and monomeric G proteins. However, extracellular effects of guanine derivatives have also been recognized. Thus, in the central nervous system (CNS), a guanine-based purinergic system that exerts neuromodulator effects, has been postulated. The thesis that GBPs are neuromodulators emerged from *in vivo* and *in vitro* studies, in which neurotrophic and neuroprotective effects of these kinds of molecules (i.e., guanosine) were demonstrated. GBPs induce several important biological effects in rodent models and have been shown to reduce seizures and pain, stabilize mood disorder behavior and protect against gliomas and diseases related with aging, such as ischemia or Parkinson and Alzheimer diseases. *In vitro* studies to evaluate the protective and trophic effects of guanosine, and of the nitrogenous base guanine, have been fundamental for understanding the mechanisms of action of GBPs, as well as the signaling pathways involved in their biological roles. Conversely, although selective binding sites for guanosine have been identified in the rat brain, GBP receptors have not been still described. In addition, GBP neuromodulation may depend on the capacity of GBPs to interact with well-known membrane proteins in glutamatergic and adenosinergic systems. Overall, in this review article, we present up-to-date GBP biology, focusing mainly on the mechanisms of action that may lead to the neuromodulator role of GBPs observed in neurological disorders.

## Purines as Intercellular Modulators

**Figure 1:**
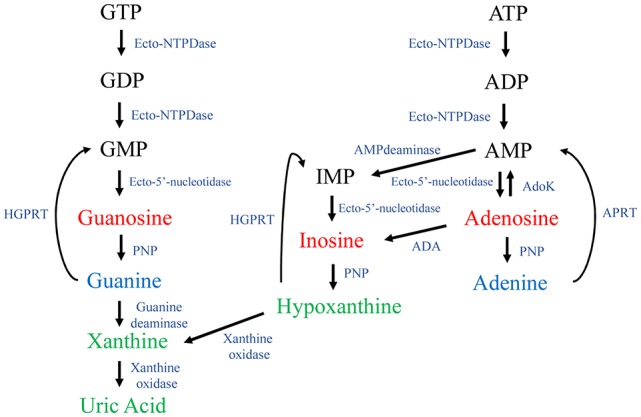
Purine metabolism. The guanine nucleotides guanosine-5′-triphosphate (GTP), guanosine-5′-diphosphate (GDP) and guanosine-5′-monophosphate (GMP) are sequentially dephosphorylated by ecto-nucleotidases (i.e., Ecto-NTPDase and Ecto-5’-nucleotidase), thus generating guanosine. Guanosine is hydrolyzed by PNP generating the purine base guanine. By action of a guanine deaminase (GDA), guanine is converted into xanthine and subsequently to uric acid by the action of a xanthine oxidase. Adenine nucleotides are also hydrolyzed forming the nucleosides adenosine and inosine. The following nitrogenous bases, hypoxanthine, xanthine and uric acid, are formed intracellularly. Ecto-NTPDase, ecto-nucleotide diphosphohydrolase or apyrase; ADA, adenosine deaminase; AdoK, adenosine kinase; APRT, adenine phosphoribosyl transferase; HGPRT, hypoxanthine-guanine phosphoribosyl transferase; PNP, purine nucleoside phosphorylase. APRT and HGPRT are mainly involved in the intracellular salvage purines pathway.

Purines are endogenous organic molecules that are essential for all cells. For instance, they are structural constituents of nucleic acids, are part of coenzyme structures and act as second messengers in intracellular signaling pathways. Purines consist of the two-ring nitrogenous bases adenine and guanine, together with the derivatives of these: nucleosides (nitrogenous bases plus a pentose sugar, usually ribose) and nucleotides (nitrogenous bases plus ribose and phosphate) that are mono-, di- or tri-phosphorylated. Adenine derivatives include the nucleoside adenosine, and the nucleotides adenosine-5′-monophosphate (AMP), adenosine-5′-diphosphate (ADP) and adenosine-5′-triphosphate (ATP). Guanine derivatives include the nucleoside guanosine, and the nucleotides guanosine-5′-monophosphate (GMP), guanosine-5′-diphosphate (GDP) and guanosine-5′-triphosphate (GTP). Purines also include some related metabolites, such as the nucleoside inosine and the nitrogenous base hypoxanthine, from the catabolism pathway of adenosine; and the bases xanthine and uric acid, from the catabolism of guanine and hypoxanthine (Figure 1). When released into the extracellular space, nucleotides are hydrolyzed by ecto-nucleotidases located at the cellular membrane surface (Zimmermann and Braun, 1996). The main ecto-nucleotide triphosphatase (ecto-NTPase) are: ecto-ATPase, which hydrolyzes ATP to ADP and GTP to GDP; ecto-ATP-diphosphohydrolase or apyrase (ecto-NTPDase), which leads to AMP from either ATP or ADP, and forms GMP from GTP or GDP (Schadeck et al., 1989). Next, the nucleosides adenosine and guanosine are the result of AMP or GMP hydrolization by ecto-5′-nucleotidase (Zimmermann, 1996). Furthermore, ecto-purine nucleoside phosphorylase (ecto-PNP) produces guanine from guanosine (Giuliani et al., 2016), while guanine deaminase (GDA) or cypin mediates guanine deamination to xanthine (Miyamoto et al., 1982; Figure 1).

The concept of purines as intercellular modulators emerged from the identification of extracellular effects of adenine-based purines (ABPs) in different cell types. Drury and Szent-Györgyi (1929) demonstrated that, after heart ischemia, adenosine is released into the extracellular space, where it promotes a negative chronotropic effect and also produces coronary vessels vasodilatation. Later, ATP was recognized to have both extracellular and intracellular effects, i.e., in the maintenance of energetic cellular metabolism (Lipmann, 1941). In the 1970s, Geoffrey Burnstock introduced the concept of purinergic nerves and purinergic neurotransmission (Burnstock et al., 1970; Burnstock, 1972). Since then, adenosine and ATP have been considered to be important neuromodulators in the central nervous system (CNS; Cunha, 2005; Jacobson and Gao, 2006; Burnstock et al., 2011; Tozaki-Saitoh et al., 2011; Ciruela et al., 2012). Meanwhile, opposed to the multiple actions reported for ABP, the importance of guanine-based purines (GBPs) has mostly been ascribed to their role as regulators of G protein function. G proteins (formerly named guanine-nucleotide regulatory proteins or GTP-binding proteins) are key to signal transduction of the so-called G protein-coupled receptors (GPCRs) to intracellular effectors (Rodbell et al., 1971). Thus, G protein activity is, up to now, modulated by the interactions with GDP or GTP in the basal and activated states, respectively (Taylor, 1990). Similarly, low molecular monomeric G protein activity is also modulated by interaction with GTP and GDP (Hepler and Gilman, 1992); while other GBPs, such as GMP, guanosine or guanine, have not been reported to interact with G proteins.

In recent years, it has been shown that, apart from the intracellular regulation of G proteins, GBPs have important extracellular effects in different tissues, especially in the CNS. Indeed, similarly to the vesicular storage reported for ATP (Gualix et al., 1999), it was found that GTP was also stored in synaptic vesicles, suggesting this guanine nucleotide would be a neurotransmitter in the brain (Santos et al., 2006). Thus, together with the existence of an adenosine-based purinergic system, a guanine-based purinergic system has been proposed (Schmidt et al., 2007). In the present review article, we report and discuss how the guanine-based purinergic system is organized in the nervous system and highlight the fundamental neuromodulator and regulatory role of these molecules.

## Extracellular Roles of Guanine-Based Purines

GBPs function in the extracellular milieu was first observed when assessing GBP levels after ischemic injury. Interestingly, it was observed that GBP were rapidly released and that their levels remain elevated until 7 days (Uemura et al., 1991). Similarly, astrocytes subjected to hypoxic or hypoglycaemic situations released different kinds of purine nucleotides, and extracellular levels of GBPs were shown to reach levels three-fold higher than those of ABPs (Ciccarelli et al., 1999). Also, it was possible to detect extracellular GBPs in human cerebrospinal fluid (CSF), both in physiological and pathological conditions (Regner et al., 1997). These findings prompted the proposal that GBPs might represent an endogenous restorative system that is activated after injury. In this context, upon brain injury, hydrolysis of the released nucleotides would occur, leading to the formation of the respective nucleosides, which may possess a protective effect.

In the CNS, the first modulatory effects of extracellular GBPs were demonstrated in the glutamatergic system (Schmidt et al., 2007), the main excitatory neurotransmission system in the brain (Meldrum, 2000; Segovia et al., 2001). Besides its essential trophic effects in the CNS, glutamate is capable of promoting a cascade of events leading to cellular death, widely known as excitotoxicity. Therefore, molecules that modulate glutamate excitotoxicity without interfering with the physiological functions of glutamate may play a fundamental role in neuroprotection. In line with this, initial studies evaluating extracellular GBP functions, indicated that guanine nucleotides would displace glutamate from its receptors (Schmidt et al., 2007; Lanznaster et al., 2016). GBPs would also modulate glutamate-induced cell responses in physiological (Tasca et al., 1995, 1998, 1999b; Regner et al., 1998; Tasca and Souza, 2000) and pathological (Regner et al., 1998; Burgos et al., 2000b) situations. Additionally, GBPs were shown to cause a delay in glutamate uptake into synaptic vesicles (Tasca et al., 2004), suggesting they modulate synaptic glutamate turnover. Moreover, other studies showed that guanosine would also modulate glutamate transporter activity (Dal-Cim et al., 2011, 2013; Lanznaster et al., 2016). In short, the identification of the extracellular actions of GBPs as intercellular messengers was possible due to five pieces of evidences: (1) GBPs can be found in the extracellular space, where they are released upon certain harmful conditions; (2) hydrolization of extracellular guanine nucleotides leads to the formation of guanosine and guanine; (3) GTP is stored in synaptic vesicles; (4) glutamate binding is displaced by guanine nucleotides; and (5) GBPs modulate glutamate transporter activity. Based on these findings, it can be concluded that GBPs act as endogenous modulators of glutamatergic transmission, and it might result in the potentially critical role that these molecules play in neuroprotection.

## Neurotrophic and Neuroprotective Effects of GBPs

### GBPs as Neurotrophic Agents

The trophic effects of GBPs, especially GTP and guanosine, in the CNS (i.e., proliferation in astrocytes and neuritogenesis in neurons) have been widely studied and are supported by many data (Neary et al., 1996; Rathbone et al., 1999; Ciccarelli et al., 2001). For example, it was first shown that GTP or guanosine treatment of cultured astrocytes promoted cell proliferation due to guanosine-induced adenosine release (Ciccarelli et al., 2001). Similarly, GTP or guanosine was also capable of stimulating the release of neurotrophic factors (i.e., fibroblast growth factor-2, FGF-2; nerve growth factor, NGF) from cultured astrocytes (Middlemiss et al., 1995; Gysbers and Rathbone, 1996a). In pheochromocytoma (PC12) cells, GTP or guanosine enhanced NGF-induced neurite arborization outgrowth (Gysbers and Rathbone, 1992, 1996b). Similarly, guanosine induced this kind of neurite arborization in PC12 cells, and also in cerebellar neurons, upon hypoxic conditions (Thauerer et al., 2010). Finally, guanosine and NGF co-treatment of PC12 cells enhanced the activity of antioxidant enzyme heme-oxygenase-1 (HO-1) and also produced an increase of cyclic GMP (cGMP) intracellular levels (Bau et al., 2005).

There are also robust findings that support the idea that GBPs play a role in neuron–astrocyte interactions. Thus, the trophic effects of GBPs were evaluated in neurons co-cultured with astrocytes, where GMP or guanosine increased the amount of cerebellar neurons and modulated the organization of the extracellular matrix proteins laminin and fibronectin in astrocytes (Decker et al., 2007). Additionally, in another work, it was demonstrated that GMP or guanosine increased the number of cultured cerebellar neurons (Tasca et al., 2010). Therefore, besides having a direct positive effect on neuronal viability, GMP or guanosine may also induce the release of soluble factors from astrocytes that favor the survival of cultured neurons. Altogether, it would be stated that GBP effects are importantly involved in neuronal migration and cell proliferation.

It should be noted that, in adulthood, neurogenesis occurs in two brain areas: the subventricular zone (SVZ) of the lateral ventricles and the subgranular zone (SGZ) in the dentate gyrus of the hippocampus (Gage et al., 1998; Gage, 2000). Indeed, in cultures of neural stem cell from the SVZ, it was recently shown that guanosine treatment increased cell proliferation and expression of brain-derived neurotrophic factor (BDNF; Su et al., 2013). Interestingly, another role for guanosine was reported. Thus, in a Parkinson’s disease (PD) mouse model, the systemic administration of guanosine stimulated neuroprogenitor cell proliferation in the SVZ (Su et al., 2009). Moreover, guanosine was also capable of increasing the number of FGF-2-positive cells, which have been shown to be critical regulators of neuroprogenitor/stem cell proliferation, survival and differentiation (Zhao et al., 2008).

### Neuroprotective Effects of GBPs

GBPs-mediated neuroprotective effects have been demonstrated using *in vivo* animal models of CNS disorders and *in vitro and ex vivo* models of excitotoxicity or oxidative damage. Here, we mainly focus on reviewing the effects of GBPs in animal models of seizures, ischemia, PD and Alzheimer’s disease (AD). Also, we review some of the related *in vitro* and* ex vivo* models of degeneration designed to identify the mechanisms of GBP-induced neuroprotection. The effects of GBPs on other CNS diseases are also briefly discussed.

#### Seizures

*In vivo* evaluation of seizures in rats or mice can be performed by using quinolinic acid, an agonist of the ionotropic glutamate receptor *N*-methyl-D-aspartate (NMDA) subtype, which causes overstimulation of glutamatergic activity (Heyes et al., 1990; Nakano et al., 1993; Meldrum, 1994). Interestingly, acute administration of GMP or guanosine reduced quinolinic acid-induced seizures by about 60% (Schmidt et al., 2000; Soares et al., 2004; Tavares et al., 2005, 2008; Torres et al., 2010). In one of those studies, the anticonvulsant effect of GMP was blocked with the 5’-nucleotidase inhibitor alpha-beta-methylene-adenosine-5’-diphosphate (AOPCP), which impedes the conversion of GMP to guanosine (Soares et al., 2004). This result suggested that guanosine is the biologically active anticonvulsant molecule. In line with this, guanosine was demonstrated to be protective both when administered intracerebroventricularly (Schmidt et al., 2005) and orally (Lara et al., 2001; de Oliveira et al., 2004); showing that its anticonvulsant effect occurs regardless the route of administration. Similarly, chronic (2 weeks) oral guanosine administration was shown to decrease quinolinic acid- and α-dendrotoxin-induced seizures (Vinadé et al., 2003, 2005). It was also shown that guanosine can modulate the changes induced in electroencephalographic (EEG) signals by quinolinic acid intracerebroventricular infusion and prevent behavioral seizures (Torres et al., 2010). Finally, in WAG/Rij rats, which is a genetic model of absence epilepsy, it was observed that guanosine decreased spike-wave discharges related. This effect was not dependent on adenosine receptor activation, since guanosine-mediated effects were not altered by using the non-selective adenosine receptor antagonist theophylline (Kovács et al., 2015).

Taken together, the data from all these studies indicate that GBPs (mainly guanosine) may modulate exacerbation of glutamatergic transmission by decreasing epileptic activity and seizures.

#### Ischemia

The most common cause of disability worldwide is brain ischemia. In the brain, blood flow reduction leads to reduced oxygen and glucose supplying, which leads to the failure of cellular bioenergetics and ultimately to excitotoxicity and oxidative stress (Durukan and Tatlisumak, 2007). GBPs-mediated neuroprotective effects can be evaluated in different brain ischemia models. One of them consists of the unilateral occlusion of the carotid artery, which is then exposed to a hypoxic atmosphere. This model is known as a perinatal hypoxia-ischemia (HI). Interestingly, guanosine was able to restore HI-induced reduction in glutamate uptake (Moretto et al., 2005, 2009). Similarly, in a model of neurological damage, such as the unilateral middle cerebral artery occlusion (MCAO), it was observed that guanosine displayed a neuroprotective effect, since it reduced the infarcted area and it also improved gait disturbances or spontaneous activity (Chang et al., 2008; Rathbone et al., 2011; Connell et al., 2013). Additionally, the neuroprotective effects of guanosine were evaluated in a rodent model of cerebral hypoperfusion (reduced blood flow) due to permanent bilateral occlusion of common carotid arteries. Interestingly, it was observed that guanosine administration (orally, for 2 weeks) reversed pyramidal neurons loss and glial fibrillary acidic protein (GFAP) expression in the hippocampus. However, guanosine did not prevent the cognitive deficit induced in this model of cerebral hypoperfusion (Ganzella et al., 2012). Another ischemic model in which the neuroprotective effects of guanosine have been shown is that induced by thermocoagulation (Hansel et al., 2014, 2015). Of note, when inducing permanent cortical focal ischemia in rats, guanosine treatment reduced the percentage of the infarcted area (around 40%). In addition, guanosine reduced neuronal degeneration, prevented reactive oxygen species (ROS) production and lipid peroxidation, which led to an improvement in forelimb dysfunction. Guanosine also reduced activation of microglia and restored the levels of inflammatory mediators (i.e., tumor necrosis factor (TNF-α), interleukins IL-1 or IL-6) in the infarcted area (Hansel et al., 2015). Importantly, these neuroprotective effects occurred when guanosine treatment was initiated just after the focal ischemia.

Apart from the data obtained from animal models, *in vitro* and *ex vivo* protocols can be useful tools to demonstrate the neuroprotective effects of GBPs in ischemic situations. For instance, brain slices subjected to oxygen and glucose deprivation (OGD) is an *ex vivo* ischemia model that allows the study of neuroprotective agents (Tasca et al., 2015). In this way, guanosine-mediated neuroprotection was observed in OGD-deprived hippocampal slices (Oleskovicz et al., 2008). Interestingly, these effects were identified to depend on the modulation of glutamate transporter activity (Dal-Cim et al., 2016), since glutamate uptake was restored to basal levels (Dal-Cim et al., 2011). Of note, guanosine would also produce antioxidant effects, since it reduced oxidative parameters (e.g., ROS production) and prevented the depolarization of the mitochondrial membrane. Interestingly, in the OGD model, guanosine has been shown to display a number of important anti-inflammatory effects. Thus, guanosine inhibited p65 (the active subunit of nuclear factor kappa B (NF-κB) transcription factor) translocation to the nucleus, or it also reduced inducible nitric oxide synthase (iNOS) expression (Dal-Cim et al., 2013). Similarly, guanosine diminished NO levels, similar to that obtained with neuronal NOS (nNOS) or iNOS isoforms inhibition (Thomaz et al., 2016).

Interestingly, glutamate challenge can also be used as an ischemic-like protocol, since ischemic events increase glutamate release and excitotoxicity. In hippocampal slices, upon glutamate-mediated damage, guanosine decreased glutamate release and prevented iNOS induction (Molz et al., 2011). Guanosine was also capable of attenuating glutamate-induced ROS production (Dalla Corte et al., 2012).

Altogether, these studies support a role for GBPs against ischemia, which may act as neuroprotective agents. Thus, GBPs would have the capacity of increasing the clearance of extracellular glutamate, prevent inflammatory events, activate antioxidant defenses and maintain mitochondria bioenergetics.

#### Parkinson’s Disease

PD is a neurodegenerative disorder, which is characterized by the selective death of dopaminergic neurons at the substantia nigra pars compacta (SNc). This loss causes important motor symptoms (i.e., bradykinesia, rigidity or postural complications; Olanow and Tatton, 1999). Several studies have evaluated the effects of GBPs in *in vivo* models of PD and the mechanisms of action of GBPs in *ex vivo* and *in vitro* models of PD.

Interestingly, guanosine was shown to decrease neuronal cell death and even produce an increase in SNc dopaminergic terminals, which ultimately led to reduce bradykinesia in a model of PD (i.e., proteasome inhibitor administration; Su et al., 2009). Another example of such neuroprotective role for guanosine was found in the PD-related alpha-synuclein A53T transgenic mouse model. Thus, this mutation led to higher guanosine brain levels when mice grew (the guanosine content would increase with aging), thus eliciting a putative protective effect (Chen et al., 2015).

Recently, the effectiveness of guanosine against dyskinesia in three rodent models of movement impairment was reported for the first time. Thus, guanosine ameliorated tremolous jaw movements (TJM) and catalepsy of reserpinized mice. In addition, guanosine potentiated contralateral rotations induced by L-DOPA in unilaterally 6-hydroxydopamine (6-OHDA)-injured rats and displayed antidyskinetic efficacy in the L-DOPA-induced dyskinesia (LID) animal model. These results support the potential usefulness of guanosine in PD management, including its capacity to reduce dyskinesia when used in combination with L-DOPA (Massari et al., 2017).

Moving to *in vitro* PD models, one of the most used is the 1-methyl-4-phenyl pyridinium (MPP^+^) model. This active metabolite of the neurotoxin 1-methyl-4-phenyl-1,2,3,6-tetrahydropyridine (MPTP) inhibits complex I activity in mitochondria (Vila and Przedborski, 2003). Notably, guanosine was able to prevent MPP^+^-induced apoptosis in SH-SY5Y neuroblastoma cells (Pettifer et al., 2007). Conversely this effect was not observed in C6 astroglial cells exposed to 6-OHDA, which is another PD model (Giuliani et al., 2016).

#### Alzheimer’s Disease (AD)

AD is a neurodegenerative condition that affects approximately 36 million people worldwide (Mazurek, 2000; Karran et al., 2011; Masters and Selkoe, 2012). Along with neuronal death, amyloid-β (Aβ) plaques and neurofibrillary tangles are the main neuropathological hallmarks of the brain of AD patients.

Different models have been used to study the neuroprotective effects of GBPs in AD. For instance, in an *in vivo* model of the pathology (intracerebroventricular infusion of Aβ_1–40_), chronic guanosine treatment prevented the cognitive deficit and anhedonic-like behavior induced in mice. Interestingly, it was shown that Aβ_1–40_ altered glutamate transport, mainly by increasing Na^+^-independent glutamate uptake, and that guanosine treatment led to recovery from this glutamatergic transmission imbalance (Lanznaster et al., 2017).

A few studies have evaluated the neuroprotective effects of GBPs in *in vitro* models of AD (i.e., using Aβ peptides). Thus, in Aβ-treated SH-SY5Y neuroblastoma cells, guanosine was able to prevent apoptosis and ROS production (Pettifer et al., 2004; Tarozzi et al., 2010). In addition, guanosine also prevented β-secretase over activity and reduced Aβ_1–42_ levels upon oxidative stress (Tarozzi et al., 2010). Similarly, in hippocampal astrocytes, guanosine prevented lipopolysaccharide (LPS)-induced inflammatory and oxidative damage as well as decreasing TNF- α and NF-κB levels by induction of HO-1 (Bellaver et al., 2015). Taken together, these data suggest that guanosine attenuates the neuroinflammation and oxidative stress induced by Aβ peptides.

#### Hepatic Encephalopathy (HE)

HE is a neurological disease that leads to cognitive impairment, which is initiated by liver dysfunction and continues with ammonia accumulation that causes alterations in extracellular glutamate levels (Butterworth et al., 1987; Albrecht and Jones, 1999). To our knowledge, only a single study has evaluated the effects of GBPs in HE. Thus, in rats subjected to bile duct ligation (BDL), guanosine was able to reverse cognitive impairment, although no changes were observed in ammonia levels. Of note, in this model oxidative stress and increased CSF glutamate levels were observed in the striatum and hippocampus, indicating that guanosine could act at this level (Paniz et al., 2014).

#### Sepsis

Sepsis, which is a potentially fatal syndrome of physiological, pathological and biochemical abnormalities associated with infection, is another condition in which GBPs may be effective. One of the existent models consists of caecal ligation and perforation. This intervention leads to oxidative stress in a number of brain regions (i.e., hippocampus, striatum, cerebellum and cerebral cortex). Interestingly, guanosine (acute administration) was shown to reduce lipid peroxidation, suggesting it would be one of the mechanisms explaining guanosine-mediated neuroprotective effects. In addition, chronic (10 days) could reduce cognitive impairment and depressive-like behavior (Petronilho et al., 2012).

#### Spinal Cord Injury

The neuroprotective effects of GBPs against spinal cord injury have also been demonstrated. In a model in which the spinal cord from rats was moderately damaged, thus leading to chronic traumatic spinal cord injury, guanosine was able to induce locomotor activity recovery (Jiang et al., 2003). Notably, guanosine treatment also led to spinal cord higher levels of bromo-deoxy-uridine (BrdU), which is a marker of cell proliferation. Thus, new mature oligodendrocytes were observed at the damaged area, which would allow remyelination and the recovery of motor activity (Jiang et al., 2003, 2008). Indeed, it is well-accepted that remyelination of the damaged area in the spinal cord is critical for functional recovery (Gilson and Blakemore, 1993; Horner et al., 2000).

#### Gliomas

Gliomas are considered the most aggressive type of brain tumors. Therapies involve surgical resection, radiotherapy or chemotherapy. One of the chemotherapeutic compounds most commonly used is temozolomide (TMZ), which is an alkylating drug that inhibits DNA replication and that has been shown to have some efficacy in early diagnosed gliomas. A few studies have analyzed the effects of GBPs on tumoral cells. Thus, guanosine was evaluated as a therapeutic strategy in several types of tumors, including lung cancer cells (Su et al., 2010), melanoma cells (Naliwaiko et al., 2008), hepatoma cells (Yang et al., 2005), leukemia and mastocytoma models (Iigo et al., 1987) and an *in vivo* Ehrlich carcinoma model (Kim et al., 1997).

In CNS tumors, the effect of guanosine was evaluated in the A172 glioma cell line. Interestingly, guanosine was cytotoxic to glioma in concentrations that were not toxic to native brain tissue (Molz et al., 2009), and to astrocytes in culture (Oliveira et al., 2017). By evaluating the combination of guanosine with the alkylating agent TMZ, it was shown that their cytotoxicity arises from the potentiation of the apoptotic process and the reduction of glioma cells migration. Guanosine also decreased mitochondrial membrane potential, intracellular ATP levels, and prevented the increase in glutamate release evoked by TMZ in glioma cells (Oliveira et al., 2017). Despite all the evidences of GBPs-mediated modulation of glutamatergic transmission, no effects of guanosine on glutamate uptake release or on the activity of glutamine synthetase have been observed (Yin et al., 2013). Additionally, the cytotoxic effect of guanosine in gliomas was not affected by glutamate receptors or transporters pharmacological blockage; while, on the other hand, adenosine receptors would be involved in guanosine-mediated cytotoxic effects (Oliveira et al., 2017). To the best of our knowledge, the anti-tumorigenic effects of guanosine in glioma cells still need further investigation. Thus, it was clearly shown that guanosine activates survival signaling pathways, which are able to induce neuroprotection or trophic effects in non-tumoral cells (Lanznaster et al., 2016). Accordingly, the cytotoxic effects of guanosine would also be related to the regulation of cell growing and survival in tumoral cells. These interesting results, together with the mechanisms that confer a dual-effect to guanosine (i.e., displaying a protective action to healthy neural cells subjected to pathological conditions and cytotoxic effect to SNC tumoral cells), need additional evaluation to be unraveled.

Overall, the studies discussed here support important neurotrophic and neuroprotective effects of GBPs, mainly GTP, GMP and guanosine. We should note that most of the neuroprotective effects observed in different pathological models were obtained using guanosine, which may be probably due to nucleotide hydrolysis occurring in the extracellular space.

## GBP Effects on Models of Pain and Mood Disorders

Anxiety, depression and pain are among the major causes of disability worldwide. Glutamate transmission has been described to be involved in the etiology and treatment of such pathologies. For instance, ketamine, which is an antagonist for NMDA glutamate receptors, was able to produce a fast-acting antidepressant effect (Chaki and Fukumoto, 2015). Furthermore, metabotropic glutamate receptor ligands have been shown to possess analgesic properties (Kolber, 2015). Considering that GBPs modulate glutamatergic transmission, it is reasonable to infer that these molecules may also present anxiolytic, antidepressant and analgesic effects.

The anxiolytic and antidepressant effects of GBPs have been studied in some well-accepted models. For instance, rats treated once with GMP or guanosine showed a decrease in anxiogenic-like behavior, which were similar to that observed with diazepam, a classic anxiolytic drug (Almeida et al., 2010). Anxiolytic-like behavior induced by guanosine was also observed after chronic treatment. Thus, guanosine (2 weeks administration) increased anxiolytic-like behavior (Da Silva and Elisabetsky, 2001; Vinadé et al., 2003). Also, a rapid antidepressant effect, similar to that of ketamine, was observed after a single oral administration of guanosine in mice (Bettio et al., 2012). Guanosine also induced antidepressant-like effects in mice subjected to acute restraint stress, a more translational model associated with mood disorders that are secondary effects of stressful lifetime events (for a review, see Yang et al., 2015). Interestingly, the antidepressant-like effects of guanosine could be due to its antioxidant activity, which would reduce hippocampal oxidative damage induced by acute restraint stress (Bettio et al., 2014). Finally, it was recently shown that chronic guanosine treatment promoted an antidepressant-like effect that might be associated with hippocampal neuroblasts differentiation (Bettio et al., 2016b), suggesting a connection between the antidepressant and neurogenic effects of guanosine (Bettio et al., 2016a).

The possible analgesic effects of GBPs have also been explored in different rodent models of pain. Guanosine reduced nociception in several acute pain models, such as acetic acid, formalin, glutamate or capsaicin injections. Guanosine treatment inhibited nociception induced by intrathecal administration of nociceptive substances, such as non-NMDA receptor agonists (Schmidt et al., 2010). Similarly, guanine inhibited nociception induced by glutamate and AMPA (de Oliveira et al., 2016). Moreover, central administration of GMP, guanosine or guanine in mice induced anti-nociception against thermal or chemical (i.e., glutamate, capsaicin) stimuli (Schmidt et al., 2008; de Oliveira et al., 2016), reinforcing the hypothesis of a CNS action of GBPs.

## Mechanisms of Action of the Protective and Trophic Effects of GBPs

As commented on above, the neuromodulatory effects of GBPs have been studied in different models of ischemia and neurodegenerative diseases, based on its action in neuroinflammation, glutamatergic toxicity and mitochondrial stress. These effects are mainly attributed to the capacity of GBPs to modulate glutamatergic transport and inhibit both oxidative stress and inflammatory damage. Meanwhile, the nucleoside guanosine, which can activate several intracellular pathways of second messengers that ultimately lead to a decrease in apoptosis, would be the main element responsible for the protective and trophic effects observed. Accordingly, most of the work aiming to elucidate the mechanisms of action of GBPs has focused on guanosine.

Several studies have demonstrated that GBP modulation of glutamate transport has a major impact on the induction of protection against excitotoxicity. Guanosine prevented glutamate release in hippocampal slices subjected to glutamatergic toxicity (Molz et al., 2011). Also, guanosine stimulated glutamate uptake both in C6 astroglial cells deprived from glucose (Quincozes-Santos et al., 2013) and in hippocampal slices subjected to OGD (Dal-Cim et al., 2013). Interestingly, these effects were all mediated by phosphatidylinositol-3 protein kinase (PI3K) and protein kinase B (Akt) pathways induction, followed by activation of mitogen-activated protein kinase/extracellular-regulated kinase (MAPK/ERK). In C6 cells, guanosine also activated protein kinase C (PKC) and p38^MAPK^ pathways to induce its protective effect. Hence, based on these studies, we can conclude that the effects of GBPs on glutamate transporters are dependent on activation of these intracellular signaling pathways. Supporting this view, a study showed that chronic administration of GMP decreased the expression of NMDA and AMPA receptor subunits and the glutamate transporters EAAC1 and GLT-1 in the rat cerebral cortex (Ganzella et al., 2012).

Regarding the ischemia and neurodegenerative models, some intracellular pathways have been identified. Thus, guanosine induced an increase in cell viability in hippocampal slices subjected to OGD, which involved the activation of several signaling pathways, including PKC, protein kinase A (PKA), MAPK/ERK and PI3K (Oleskovicz et al., 2008). PI3K activation is also involved in the anti-apoptotic effect of guanosine in cultured human neuroblastoma cells challenged with β-amyloid, which may include an increase in phospho-Akt (Pettifer et al., 2004). Similarly, Akt expression and activation was increased by guanosine, which protected astrocytes challenged with the apoptotic drug staurosporine (Di Iorio et al., 2004). Also, guanosine activated the PI3K/Akt/GSK3β pathway, which inhibits the oxidative damage produced by blocking mitochondrial complexes I and V (Dal-Cim et al., 2012). Furthermore, in 6-OHDA-treated SH-SY5Y cells, a model for PD, guanosine reduced p38^MAPK^ and Jun Kinase (JNK) activation, and increased phospho-Akt and the expression of the anti-apoptotic protein, Bcl-2 (Giuliani et al., 2012b). Finally, guanosine also activated the PI3K pathway, which promotes anti-inflammatory effects. It also inhibited iNOS induction via PI3K and MAPK/ERK activation in hippocampal slices subjected to OGD (Dal-Cim et al., 2013).

Notably, the involvement of the PI3K/Akt pathway in GBP mechanisms of action has also been reported for the antidepressant effects of guanosine. In addition, guanosine-mediated antidepressant effects would also depend on the activation of the mammalian target of rapamycin (mTOR; Bettio et al., 2012).

Finally, regarding GBP-induced trophic effects, similar signaling pathways have been reported. Guanosine promoted neurite outgrowth in primary cerebellar neurons culture by activation of PCK-related kinase-1 (PRK1; Thauerer et al., 2010), which is a kinase involved in neuronal differentiation (Manser et al., 2008). Similarly, by increasing cAMP accumulation, phosphorylation of cAMP response element-binding protein (CREB), and BDNF mRNA levels, guanosine induced neural stem cell proliferation (Su et al., 2013). ERK, CaMKII, PKC, PI3K and PKA were also involved in the trophic effect of guanosine in cultured cerebellar neurons (Tasca et al., 2010). Indeed, the effects of guanosine in the MAPK/ERK, CaMKII, PKC, PI3K and PKA signaling pathways were observed to correlate with the reorganization (from a diffuse to a fibrillary matrix) of extracellular matrix proteins (Decker et al., 2007).

**Figure 2:**
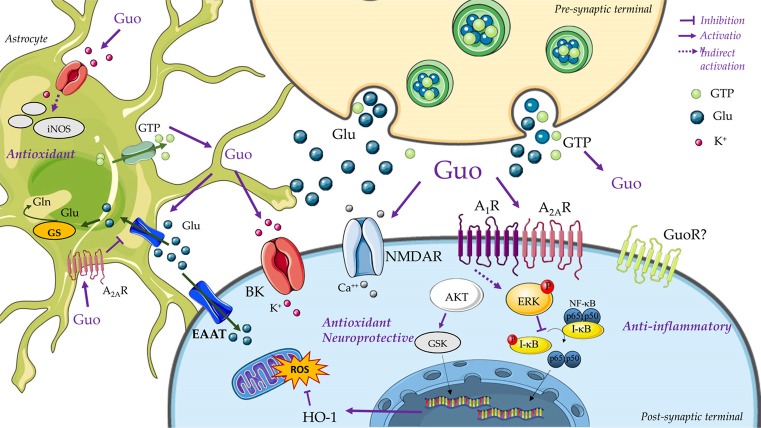
Guanine-based purinergic signaling. This cartoon presents an overview of the main mechanisms involved in the neuroprotective effects of guanosine. The release of guanine nucleotides (i.e., GTP) from synaptic vesicles or astrocytes produces extracellular guanosine (Guo) following hydrolysis by ecto-nucleotidases. Guanine may also be formed by PNP activity on guanosine (not represented). A specific binding site for Guo has not been disclosed yet (i.e., guanosine receptor, GuoR), but guanosine effects through calcium-dependent (big) conductance potassium (BK) channels, A_1_R and A_2A_R adenosine receptors, and glutamatergic N-methyl-D-aspartate receptors (NMDARs) has been described. Guanosine promotes neuroprotection through an anti-inflammatory effect of inhibiting nuclear factor kappa B (NF-κB) activation via MAPK/ERK. Additionally, guanosine presents antioxidant effects, as reducing the reactive oxygen species (ROS) generation, preventing inducible nitric oxide synthase (iNOS) expression, and by increasing antioxidant defenses, as Heme-oxygenase-1 (HO-1) levels. Activation of PI3K/Akt, protein kinase C (PKC) and MAPK/ERK by guanosine leads to stimulation of excitatory amino acid transporters (EAATs) activity. Guo also increases glutamine (Gln) synthetase (GS) activity, thus reducing extracellular levels of glutamate (Glu) and protecting from glutamate excitotoxicity. Figure designed using image templates from Servier Medical Art https://smart.servier.com/image-set-download/.

Overall, GBPs have been shown to activate several intracellular pathways that promote biological effects (Figure 2). It could then be concluded that the PI3K pathway is one of the main intracellular routes involved in guanosine-induced neuroprotection. Importantly, the identification of GBP receptor(s), which is discussed below, will help to determine the exact sequence of cell signaling pathways activated by GBPs.

## GBP Receptors: Are GBPs Orphan Molecules?

Selective binding sites for GBPs have already been confirmed in rat brain membranes (Traversa et al., 2002, 2003); but GBP receptors have not yet been identified. The existence of exclusive protein receptors for GBPs are mainly supported by the use of [^3^H]guanosine in rat brains. Accordingly, it was possible to identify a single high-affinity guanosine binding site (dissociation constant (*Kd*): 95.4 ± 11.9 nM; and apparent number of maximal binding sites (Bmax): 0.57 ± 0.03 pmol/mg protein). Other GBP and guanine metabolites were not potent displacers of guanosine binding, strengthening the hypothesis that guanosine is the ultimate GBP with biological effects. Nevertheless, recent studies also highlighted biological effects for guanine, mainly through modulating memory and learning impairment (Giuliani et al., 2012a; Zuccarini et al., 2018).

One of the hypotheses to identify GBP (or guanosine) receptors consists of their interaction with adenosine receptors. However, given that adenosine, together with caffeine or theophylline (non-selective adenosine receptor antagonists) were not capable of displacing guanosine binding, it would seem likely that other receptors may be involved. Indeed, in 2011, it was identified a new putative GPCR that could be selectively activated by guanosine (Volpini et al., 2011). On the other hand, it would also seem likely that guanosine-mediated effects depend on Gα_i_-protein activation. In this way, by means of the pertussis toxin (PTX), an inhibitor of Gα_i_ family proteins, it was possible to reduce guanosine binding by 45% (Traversa et al., 2003). Interestingly, other studies have focused on assessing whether GBPs could also interact with transporters in the purinergic system. Thus, it was found that a nucleoside transporter inhibitor (nitrobenzylthioinosine) and an inhibitor of adenosine reuptake (propentofylline) had no effect on guanosine binding (Traversa et al., 2003).

Although the identification of putative GBP receptors is a major goal, research has also focused on elucidating the role of some well-characterized membrane proteins, such as glutamate receptors and transporters, adenosine receptors and potassium channels, which have been shown to be involved in the effects of GBPs. The diverse findings suggest that GBPs, especially the nucleoside guanosine, may act as multi-target neuromodulators (Lanznaster et al., 2016).

### Glutamate Transporters or Receptors

As discussed above, GBPs can modulate the activity of the glutamatergic system; therefore, a possible direct interaction with glutamate receptors has been explored. Initial binding studies showed that GBPs can displace the binding of glutamate and its analogs to receptors in cell membrane preparations (Sharif and Roberts, 1981; Butcher et al., 1986; Monahan et al., 1988; Baron et al., 1989; Hood et al., 1990; Paas et al., 1996). It was also shown that GBP-induced glutamate displacement would not depend on the interaction with G proteins or on the reduction of agonist binding to other GPCRs (Souza and Ramírez, 1991; Paz et al., 1994; Ramos et al., 1997; Porciúncula et al., 2002; Rotta et al., 2004). Reinforcing this hypothesis, several studies showed that GMP is capable of decreasing glutamate binding to receptors that do not interact with G proteins (Burgos et al., 1998, 2000a,b; Aleu et al., 1999; Tasca et al., 1999a; Tasca and Souza, 2000). Meanwhile, a number of studies failed to observe a direct interaction of other GBPs with glutamate receptors; guanosine and guanine had no effect on the binding of glutamate and its analogs in total rat brain membranes (Traversa et al., 2002; Vinadé et al., 2003). Binding experiments with the post-synaptic density-enriched fraction showed that GppNp (a poor hydrolyzable GTP analog) and GMP displaced 40% and 36%, respectively, of glutamate binding, while guanosine only displaced 23%. Similarly, AMPA binding was not affected by guanosine, but was inhibited 21% and 25% by GppNp and GMP, respectively (Porciúncula et al., 2002).

Considering the capacity of GBPs to modulate glutamate transport, they could also interact with glutamate transporters. Interestingly, the enhancement of glutamate uptake by guanosine was first identified (Dal-Cim et al., 2011, 2013) together with the fact that guanosine reduced glutamate release (Molz et al., 2011). More recently, it was shown that synthetic glutamate transporter inhibitors also inhibited the decrease in glutamate release promoted by guanosine in an *in vitro* ischemia model (Dal-Cim et al., 2016); suggesting a direct interaction of guanosine with glutamate transporters. Nevertheless, despite these data, a direct guanosine-glutamate transporter interaction has still not been demonstrated.

### Adenosine Receptors

To date, the involvement of the adenosinergic system in guanosine-mediated effects is still a matter of debate. As discussed above, binding studies seemed to rule out a direct interaction of guanosine with adenosine receptors. However, several biological effects induced by guanosine seem to be dependent on adenosine receptors, namely on A_1_R and A_2A_R. For example, although caffeine (a non-selective adenosine receptor antagonist) did not alter guanosine binding in rat brain (Traversa et al., 2003), it reversed the guanosine-mediated anxiolytic-like behavior. In contrast, caffeine was not able to block the effects of guanosine in capsaicin-induced nociception (Schmidt et al., 2008), and the anticonvulsant effect of guanosine on QA-induced seizures in mice (Lara et al., 2001). Similarly, DPCPX, a selective A_1_R antagonist blocked guanosine anti-nociceptive effects, but not SCH58361, an A_2A_R antagonist (Schmidt et al., 2008). Contradictorily, in cultured cerebellar neurons, the trophic effects of guanosine depended on A_2A_R, since the selective antagonist ZM241385 blocked increased neuronal adhesion mediated by guanosine (Tasca et al., 2010).

The involvement of A_1_R and A_2A_R in the guanosine cytoprotective effects has been well characterized in *in vitro* and *ex vivo* studies. In a human neuroblastoma cell line (SH-SY5Y) subjected to mitochondrial oxidative stress, the protective effects of guanosine were blocked by DPCPX and ZM241385 (Dal-Cim et al., 2012). Guanosine induced a decrease in ROS formation and mitochondrial membrane potential in hippocampal slices subjected to OGD, which was dependent on A_1_R, since DPCPX treatment inhibited the protective effect of guanosine; however, A_1_R inhibition did not affect the effect of guanosine on glutamate uptake. Also, PTX blocked the effects of guanosine mediating recovery from OGD-induced glutamate uptake impairment, suggesting an interaction with a Gα_i_ protein-coupled receptor. In contrast, activation of A_2A_R by agonist CGS21680 inhibited guanosine-induced neuroprotection, but A_2A_R blockade with the antagonist ZM241385 did not; both assessed by cellular viability and glutamate uptake (Dal-Cim et al., 2013). These controversial results support the hypothesis that guanosine needs the presence of both A_1_R and A_2A_R, which could be related to the fact that these receptors directly interact forming the so-called receptor oligomers. Indeed, it was demonstrated that these receptors associate in the membrane of cells and interact with each other in an antagonistic manner (Ciruela et al., 2006a,b; Ciruela, 2013). Further studies should be performed to demonstrate this hypothesis.

### Potassium Channels

Apart from its effects on GPCRs commented on above, guanosine has also been shown to modulate the activity and expression of K^+^ channels. In this way, the function of inward rectifier K^+^ channels was increased upon guanosine challenge in cultured rat cortical astrocytes (Benfenati et al., 2006). Also, the neuroprotective effects of guanosine would also depend on K^+^ channel activity. In line with this, charybdotoxin, which blocks large-conductance Ca^2+^-activated K^+^ channels (BK), was able to impede guanosine-mediated cellular viability increase in OGD-treated hippocampal slices and also blocked the protective effects of guanosine on SH-SY5Y cells subjected to mitochondrial damage (Dal-Cim et al., 2011, 2012). BK blockage also inhibited guanosine-mediated recovery of the decreased glutamate uptake in hippocampal slices subjected to OGD (Dal-Cim et al., 2011). It should be noted that other potassium channels do not seem to modulate guanosine-mediated neuroprotective effects. Thus, guanosine-induced neuroprotection was not affected by the inhibition of ATP-sensitive K^+^ channels (with glibenclamide) or small-conductance Ca^2+^-activated K^+^ (SK) channels (with apamin; Dal-Cim et al., 2011). Indeed, guanosine increased K^+^ conductance in cells transiently transfected with the functional α-subunit of BK channels but not with SK channels (Tasca et al., 2013).

From the reports discussed here, it seems clear that additional studies are necessary to unravel the putative molecular targets of guanosine (and even of guanine), in order to understand the actual complexity of the GBP system.

## Perspectives on the Guanine-Based Purinergic System

GBPs exert a plethora of beneficial (i.e., neuroprotective, neurotrophic, antidepressant, anxiolytic and analgesic) effects throughout the CNS. Accordingly, in recent years, much effort has been devoted to fully characterize these kinds of effects and elucidating the still unresolved mechanisms of action of these molecules. Importantly, the lack of identification of putative GBP receptors, which may mediate some of the observed effects (other receptors, such as glutamate or adenosine receptors, and ion channels, may also be involved) has slowed this process. For this reason, in the next few years, an exponential rise in GBP studies is expected, in which the elucidation of both the mechanism of action of these molecules and the exact role they play in some pathologies (e.g., AD and PD) may be the main goals. Meanwhile, some empirical data can already be successfully used in a number of clinical situations. For instance, drugs facilitating the salvage pathway of purine recycling, such as allopurinol, a xanthine oxidase activity inhibitor, could be included as an option for refractory epilepsy (Togha et al., 2007). Similarly, alteration of purine metabolism may be considered a possible biomarker for PD diagnosis. Thus, a reduction in uric acid plasma levels can be observed in PD patients (Schwarzschild et al., 2008), while increased urate levels have been found in post-mortem PD brains. Altogether, these data reinforce the notion that purine levels and metabolism may modulate response of the organism to injury, acting as a retaliatory system responsible for modulating unbalanced neurotransmission in damaging situations. Overall, together with evidences showing that GBPs may have both trophic effects on neural cells and beneficial behavioral effects, present knowledge of GBP biology in the CNS supports the rationale for further studies of this system and the development of novel drugs that may be useful in the near future.

## Author Contributions

CT wrote the article and made illustrations. DL, KO, VF-D and FC wrote the article.

## Conflict of Interest Statement

The authors declare that the research was conducted in the absence of any commercial or financial relationships that could be construed as a potential conflict of interest.
